# Role of multiparametric magnetic resonance imaging in early detection of prostate cancer

**DOI:** 10.1007/s13244-016-0466-9

**Published:** 2016-02-04

**Authors:** Pieter J. L. De Visschere, Alberto Briganti, Jurgen J. Fütterer, Pirus Ghadjar, Hendrik Isbarn, Christophe Massard, Piet Ost, Prasanna Sooriakumaran, Cristian I. Surcel, Massimo Valerio, Roderick C. N. van den Bergh, Guillaume Ploussard, Gianluca Giannarini, Geert M. Villeirs

**Affiliations:** Department of Radiology, Ghent University Hospital, De Pintelaan 185, 9000 Ghent, Belgium; Department of Urology, Urological Research Institute, Vita-Salute University San Raffaele, Milan, Italy; Department of Radiology and Nuclear Medicine, Radboud UMC, Nijmegen, The Netherlands; Department of Radiation Oncology, Charité Universitätsmedizin Berlin, Berlin, Germany; Department of Urology, Regio Clinic Wedel, Wedel, Germany; Department of Urology, University Hospital Hamburg-Eppendorf, Hamburg, Germany; Department of Oncology, Institut Gustave Roussy, University of Paris Sud, Villejuif, France; Department of Radiation Oncology and Experimental Cancer Research, Ghent University Hospital, Ghent, Belgium; Surgical Intervention Trials Unit, Nuffield Department of Surgical Sciences, University of Oxford, Oxford, UK; Department of Molecular Medicine & Surgery, Karolinska Institutet, Stockholm, Sweden; Centre of Urological Surgery, Dialysis and Renal Transplantation, Fundeni Clinical Institute, Bucharest, Romania; Department of Urology, CHUV, Lausanne, Switzerland; Department of Urology, Radboud University Nijmegen Medical Centre, Nijmegen, The Netherlands; Urology Department, Saint Jean Languedoc Hospital, Toulouse, France; Research Unit INSERM U955, Paris Est University, Team 7, Paris, France; Urology Unit, Academic Medical Centre Hospital «Santa Maria della Misericordia», Udine, Italy

**Keywords:** Prostate, Prostatic neoplasms, Magnetic resonance imaging, Magnetic resonance spectroscopy, Diffusion magnetic resonance imaging

## Abstract

**Abstract:**

Most prostate cancers (PC) are currently found on the basis of an elevated PSA, although this biomarker has only moderate accuracy. Histological confirmation is traditionally obtained by random transrectal ultrasound guided biopsy, but this approach may underestimate PC. It is generally accepted that a clinically significant PC requires treatment, but in case of an non-significant PC, deferment of treatment and inclusion in an active surveillance program is a valid option. The implementation of multiparametric magnetic resonance imaging (mpMRI) into a screening program may reduce the risk of overdetection of non-significant PC and improve the early detection of clinically significant PC. A mpMRI consists of T2-weighted images supplemented with diffusion-weighted imaging, dynamic contrast enhanced imaging, and/or magnetic resonance spectroscopic imaging and is preferably performed and reported according to the uniform quality standards of the Prostate Imaging Reporting and Data System (PIRADS). International guidelines currently recommend mpMRI in patients with persistently rising PSA and previous negative biopsies, but mpMRI may also be used before first biopsy to improve the biopsy yield by targeting suspicious lesions or to assist in the selection of low-risk patients in whom consideration could be given for surveillance.

***Teaching Points*:**

• *MpMRI may be used to detect or exclude significant prostate cancer*.

• *MpMRI can guide targeted rebiopsy in patients with previous negative biopsies*.

• *In patients with negative mpMRI consideration could be given for surveillance*.

• *MpMRI may add valuable information for the optimal treatment selection*.

## Introduction

Most prostate cancers (PC) are currently found on the basis of an elevated serum prostate specific antigen (PSA) level. At present, PSA is the best standard biomarker used for early detection of PC [1–4], although it has only moderate sensitivity (PC may still be present at low levels of PSA) and specificity (benign prostatic hyperplasia or prostatitis may cause false positive results) [1, 2, 5, 6]. In patients with elevated PSA, histological confirmation is needed and this is traditionally obtained using random transrectal ultrasound (TRUS) guided prostate biopsy [7]. This approach, however, yields false negative results in up to 40 % of cases and may show underestimation of Gleason grade, especially in anteriorly located tumours [8–10]. Treatment selection is based on prognostic factors including serum PSA level, histological grading (Gleason score), tumour size, clinical staging (TNM), and patient’s life expectancy [1, 4, 5]. Population-based PC screening with PSA is discouraged by international guidelines, because of potential overdiagnosis and subsequently overtreatment of non-lethal disease [11, 12].

The implementation of multiparametric magnetic resonance imaging (mpMRI) into a screening program currently seems to be the most promising technique to reduce the risk of overdetection of non-significant PC and improve the early detection of clinically significant PC [13]. The role of mpMRI has evolved in the past decade. The EAU, NCCN, and ESUR guidelines currently recommend the use of mpMRI in patients with persistently rising PSA and previous negative biopsies in an attempt to visualize the PC and consequently make targeted rebiopsy possible [13–17]. New MRI sequences and scanning techniques have boosted the diagnostic accuracy of mpMRI and its potential to be used as an additional decision tool before first biopsy to improve the biopsy yield or to assist in the selection of men who might reasonably defer unnecessary biopsy [5, 13, 18, 19].

In this paper we give an overview of the mpMRI technique, its performance in PC detection, and in the assessment of tumour aggressiveness and size. The role of mpMRI in early detection of clinically significant PC is highlighted.

## Clinical significance of a PC

It is generally accepted that a PC with a Gleason score of ≥4 + 3 and/or non-organ confined disease is clinically significant and requires treatment depending on patients’ life expectancy [20, 21]. Substantial recent data suggest that a small PC with pure Gleason 3 + 3 does not pose a significant threat to a man’s life allowing deferment of treatment and inclusion in an active surveillance program as a valid option [22, 23]. There is more discussion among urologists about the clinical significance of a large Gleason 3 + 3 PC or a Gleason 3 + 4 PC with limited grade 4 (less than 10 %) [20, 22, 24]. Epstein et al. recently proposed a new PC grading system based on the Gleason scores with grade group 1 including all Gleason score 6, grade group 2 including Gleason score 3 + 4, grade group 3 Gleason score 4 + 3, grade group 4 Gleason score 8, and grade group 5 Gleason score 9–10 PC. This simplified grading system avoids the logical yet incorrect assumption for patients with a Gleason score 6 PC that their cancer is in the middle of a scale of 2 to 10 (from Gleason score 1 + 1 to Gleason score 5 + 5), potentially reducing anxiety and overtreatment for indolent disease [25]. Further, this novel grading system highlights the different prognosis of men with Gleason score 7, according to the presence of a primary or a secondary pattern 4.

## Multiparametric magnetic resonance imaging

A mpMRI consists of morphological T2-weighted images (T2-WI) supplemented with functional imaging techniques such as diffusion-weighted imaging (DWI), dynamic contrast enhanced imaging (DCE), and/or magnetic resonance spectroscopic imaging (MRSI) [14]. This results in a combination of anatomical, biological, and functional information.

On T2-WI the prostatic morphology is depicted with high resolution. The transition zone (TZ) of the prostate consists of nodular areas of stromal and glandular hyperplasia with varying signal intensity (SI), and is surrounded posteriorly and laterally by the peripheral zone (PZ) that usually has a more homogeneous high SI [26, 27]. PC can be identified on T2-WI as an ill-defined low SI area, contrasting well with the normal high background SI of the PZ, but often more difficult to distinguish in the TZ [28, 29]. The reported sensitivities and specificities for detection of PC with T2-WI range between 57 %-88 % and 28 %-94 %, respectively [18, 28, 30–41]. The lower specificities may be explained by diseases with low SI areas in the PZ mimicking PC. Nevertheless, T2-WI has been assumed to be the dominant sequence for the identification of PC in the TZ when interpreting imaging findings of a mpMRI exam [42, 43].

DWI provides information about the amount of random movement of water molecules in a tissue [44, 45]. In healthy prostate glands the water molecules move relatively unhindered but in PC the motion is strongly inhibited. This is depicted on DWI as a high SI area on high-b-value images with a corresponding low apparent diffusion coefficient (ADC) [44–47]. The reported sensitivities and specificities of DWI for detection of PC range from 57-93 % and 57-100 %, respectively [48, 49]. DWI has been observed to be the best-performing single parameter for detection of PC in the PZ deserving the strongest weighting in a mpMRI exam [43, 50].

In DCE, the prostate is evaluated on serial T1-weighted images after an intravenous bolus injection of contrast agent [5, 51]. The shape of the dynamic enhancement curve depends on vascular permeability in the examined tissue. Most PC are associated with neoangiogenesis and increased vascular permeability resulting in pronounced contrast enhancement and a curve showing high peak enhancement and early washout [5, 51]. Alternatively, several quantitative post-processing perfusion MRI parameters have been developed, such as K_trans_, v_e_ and k_ep_ [45, 51, 52], but currently the localization of the enhancement (focal or diffuse and whether or not corresponding to suspicious findings on T2 and/or DWI) as compared to the adjacent normal prostatic tissue is considered more important than the enhancement characteristics of the lesion itself. [53] Accuracies of 70 to 90 % have been reported for DCE in detection of PC [51, 52]. Mowatt et al. showed a pooled sensitivity of 79 % (69 %-87 %) and a pooled specificity of 52 % (14 %-88 %) [41]. False positive enhancement in benign prostatic hyperplastic nodules in the TZ or in prostatitis in the PZ causes overlap in enhancement patterns between tumours and benign conditions yielding lower specificity [54].

MRSI provides information about the relative concentrations of cellular metabolites in the prostate such as citrate and choline [5, 44, 55]. Citrate is synthesized, stored, and secreted by healthy prostatic glandular tissue, and choline is an important constituent in the cell membrane metabolism and its concentration increases in highly cellular areas such as in PC [55, 56]. The complimentary changes of both metabolites are used to predict the presence or absence of PC. Diagnostic accuracies up to 70-90 % for detection of PC with MRSI have been reported [44, 56–59]. Mowatt et al. showed a pooled sensitivity of 92 % (86 %-95 %) and pooled specificity of 76 % (61 %-87 %) [41].

A mpMRI is preferably performed according to the uniform quality standards of the European Society of Urogenital Radiology (ESUR) guidelines [14]. For reporting prostate mpMRI, many authors in the past have used a subjective 5-point Likert score to communicate the conclusion to the referring clinician, but in 2012 the ESUR introduced the Prostate Imaging Reporting and Data System (PI-RADS) in analogy with the Breast Imaging Reporting and Data System (BI-RADS) [14, 60, 61]. The PI-RADS reporting system entails assignment of separate scores for each of the scanned mpMRI sequences and provides explicit verbal descriptions on how to generate them. Each exam is assigned with an overall assessment score ranging from 1 (indicating that clinically significant PC is highly unlikely to be present) to 5 (indicating that clinically significant PC is highly likely to be present) (Table 1). In 2015 a modified version of PI-RADS was published, named PI-RADS version 2 (PI-RADSv2), which was also adopted by the American College of Radiology (ACR) [53]. The previous version of PI-RADS was from then on referred to as PI-RADS version 1 (PI-RADSv1). In PI-RADSv1 the overall assessment category score was based on a subjective radiologist’s impression of the scanned sequences, but in PI-RADSv2 two dominant modalities have been defined, namely, DWI for the peripheral zone (PZ) and T2-WI for the TZ, and well-defined instructions have been provided on how to determine the overall assessment score [43, 53, 62, 63].

**Table 1:** The PIRADS overall assessment categories

PIRADS 1	Clinically significant prostate cancer is highly unlikely to be present
PIRADS 2	Clinically significant prostate cancer is unlikely to be present
PIRADS 3	Clinically significant prostate cancer is equivocal
PIRADS 4	Clinically significant prostate cancer is likely to be present
PIRADS 5	Clinically significant prostate cancer is highly likely to be present

## Performance of mpMRI

Validation studies of mpMRI are accumulating and show that this imaging technique provides high detection rates of PC, but the location of the tumour in the prostate (PZ or TZ), the volume, and histological characteristics such as Gleason score highly influence its performance [64–66]. The reported accuracy of mpMRI at detecting PC varies with the definition of clinically significant PC, and the mpMRI threshold that is used (i.e., whether PI-RADS 3 is considered positive or negative) [67, 68]. With mpMRI, sensitivities and specificities of 71-84 % and 33-70 %, respectively, are reported for detection of PC of any grade, and 80-90 % and 47-61 %, respectively, for detection of high-grade (HG) PC [28, 69–80]. A recently published systematic review reported accuracies of 44-87 %, sensitivities of 58-97 %, and specificities of 23-87 % for detection of clinically significant PC using mpMRI, with trends depending highly on the threshold used in the definition of clinically significant disease [81]. A meta-analysis of 14 studies evaluating PI-RADS reported a pooled sensitivity of 78 % (95 % CI 70 %-84 %) and pooled specificity of 79 % (95 % CI 68 %-86 %) for detection of PC with mpMRI [82]. Abd-Alazeez et al. reported that, when a PI-RADS overall assessment score of 3 was used as threshold for a positive mpMRI, 100 % sensitivity and 100 % NPV was achieved for detection of Gleason 4 + 3 PC, at the expense of 19 % specificity due to many false positives. When a PI-RADS overall assessment score of 4 was used as a threshold for the detection of Gleason 4 + 3 PC, specificity increased to 61 % (indicating less false positives), with still having a sensitivity of 92 % and a NPV of 99 % [68].

## Assessing tumour aggressiveness and size with mpMRI

The histological Gleason score is a critical predictor of PC aggressiveness, and correlations have been reported between PC Gleason score and T2, DWI, and MRSI suggesting that mpMRI may be able to non-invasively assess PC aggressiveness [5, 55, 57, 64–66, 83, 84]. On DWI, lower ADC values are strongly correlated with higher Gleason scores [44–46, 85–89]. On T2-WI, lower SI of a PC relative to muscle seems to be associated with higher Gleason scores [90] and on MRSI the relative concentrations of citrate and choline tend to correlate with Gleason scores [57, 59, 91]. Higher grade PC are histologically associated with more pronounced destruction of the normal ductular system, more solid areas of tumour cells and less fluid content as compared to lower grade PC, and, consequently, show a higher detectability on mpMRI [64–66, 92].

On mpMRI, tumour size can be measured on high-resolution axial, sagittal, and coronal T2-WI planes. Assessment of size is easier when a lesion is sharply demarcated than when it is ill-defined, but size estimates show considerable inaccuracy with a little more overestimation than underestimation [72]. Very small tumours <1 mm diameter are below the detection limit of mpMRI, but a PC of 1 cm diameter (0.5 ml) is well within the 3 or 4 mm spatial resolution of T2-WI [64–66, 92, 93]. PC volume thus influences its detectability on mpMRI with larger tumours being detected more easily than smaller ones. While the prognostic value of tumour volume is questionable because the largest foci may not necessarily represent the biologically most significant tumours, small PC foci are often regarded as clinically insignificant. Historically, a tumour volume of less than 0.5 ml has been proposed as the threshold for clinical significance [24, 94]. Recent studies estimate that after accounting for tumour stage (maximum pT2) and tumour grade (maximum Gleason score 6), a volume threshold at 1.3 ml or even 2.5 ml may be applied [22, 24]. Moreover, most PC are multifocal, and controversy exists about whether the total tumour volume in the prostate should be taken into account, or only the volume of the largest dominant (index) lesion [7, 24, 95–97].

PC detection rates on mpMRI, thus,depend highly on the Gleason grade and size of the tumour, ranging from 21-29 % for <0.5 ml tumours with Gleason 6 to 100 % for tumours >2 ml with Gleason ≥8 [64]. MpMRI detects both higher grade and larger PC more accurately suggesting that it may perform particularly well for the detection of clinically significant disease.

## Clinical role of mpMRI in early detection of PC

Until a few years ago, mpMRI of the prostate was mainly used for staging purposes after histological confirmation of PC, but in recent years a re-evaluation of its position is going on. The ability of mpMRI to selectively detect higher grade and larger volume tumours may help to discriminate between significant cancer from indolent cancer, which is useful in the initial assessment of men considering active surveillance [98], or it may exclude significant cancer before biopsy, which might lead to a refined diagnostic pathway. The information obtained with mpMRI is potentially useful as an additional tool in screening protocols for the assessment of patients with elevated PSA, next to clinical risk stratification variables such as digital rectal examination, patient’s age, ethnicity, comorbidity, and family history. MpMRI may have value in men with elevated PSA to help identify areas in the prostate with high probability of being cancerous to improve the biopsy yield, and it may even serve as a triage test to determine which patient should undergo prostate biopsy or in whom a biopsy may be deferred [96]. The benefits of incorporating mpMRI into the diagnostic algorithm may outweigh its costs by preventing unnecessary biopsies and reduce overtreatment when mpMRI is negative, and by improving the characterization of PC using (only) targeted biopsy to a suspicious lesion when mpMRI is positive [67, 72, 96, 99–104]. Moreover, when mpMRI is performed before biopsy, hemorrhagic post-biopsy artefacts [105] are avoided, and, in case of a positive diagnosis of PC, the imaging is immediately available for staging [68, 96]. The current PC guidelines do not generally include mpMRI before initial biopsy, but the arguments for scanning before biopsy become stronger. The systematic review of Moore et al. [106] showed that with pre-biopsy mpMRI and targeted MRI-guided biopsy an equal number of clinically significant PC was detected (in 43 % of the presenting population) as compared with standard random TRUS biopsy, but a third fewer men were biopsied overall, a greater proportion of men with clinically significant PC was biopsied, and 10 % fewer men were attributed a diagnosis of clinically insignificant PC. In the more recent systematic review of Schoots et al. [107] MRI-targeted biopsy (and omitting the biopsy when mpMRI is negative) showed similar overall PC detection rates (sensitivity of 0.81 vs 0.85), higher detection rates of clinically significant PC (sensitivity of 0.91 vs 0.84), and lower (unwanted) detection rates of insignificant PC (sensitivity of 0.44 vs 0.83) as compared to random TRUS-guided biopsy. Subgroup analysis revealed especially an improvement in PC detection in men with previous negative biopsy than in biopsy-naïve men (relative sensitivity of 1.54 vs 1.10 for clinically significant PC and 1.62 vs 0.97 for any PC) [107]. Tonttila et al. [108] however recently performed a randomized prospective blinded controlled trial and concluded that adding mpMRI before biopsy did not improve PC detection rates as compared to TRUS biopsy alone in patients with elevated PSA. They reported that adding mpMRI elevated the detection rate from 57 % to 64 % for any PC and from 45 % to 55 % for clinically significant PC, but both differences were not statistically significant. This study was, however, performed in a hospital-based practice setting instead of a centre of excellence for prostate mpMRI, which was reflected in mpMRI that were not scanned or reported according to the PI-RADS standards and limited experience of the urologists who performed targeted biopsies using a cognitive approach, which may all be considered major limitations that will have negatively influenced the results [108].

## Targeted biopsy when mpMRI is suspicious

**Fig. 1:**
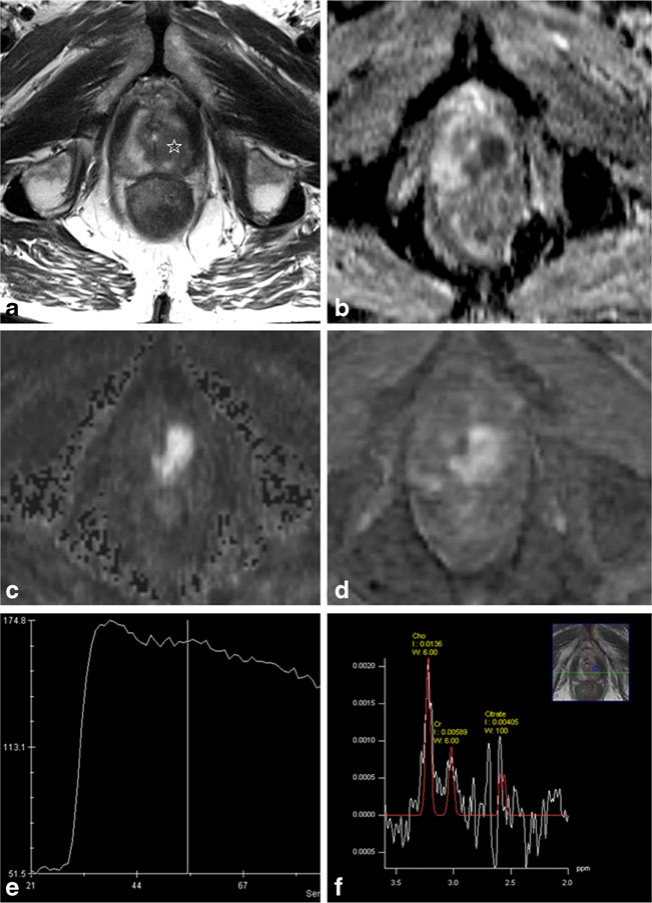
67-year-old man with persistently elevating PSA up to 6 μg/l. On this mpMRI a very suspicious lesion is detected in the apex of the prostate at the left side (white star). Morphologically it is demonstrated as an area of low SI with irregular margins on T2-WI (**a**), low ADC value (**b**) with high SI on the high-b-value image (**c**) of the DWI, strong contrast enhancement (**d**) with early and high peak enhancement with wash-out on the DCE curve (**e**). On MRSI the citrate peaks are reduced and the choline peaks elevated. The overall assessment score of this exam was PI-RADS 5. A targeted TRUS-biopsy with knowledge of the mpMRI findings (i.e., a MR-guided biopsy with cognitive fusion) confirmed a Gleason 4 + 5 PC in the apex at the left side

An accurate prostate biopsy is an important component in the decision-making and treatment selection. A suspicious mpMRI (PI-RADS overall assessment score 4 and 5) enables image-guided targeted sampling at the suspicious areas to overcome the limitations of the traditional blind systematic prostate biopsy. MR-guided biopsies are becoming more and more available, but there is currently no consensus on the optimal technique. [17] In-bore MR-guided biopsy is accurate, but requires considerable technical requirements, which are lacking at most centres, and has significant cost and logistic issues [17, 109]. As an alternative, suspicious lesions on mpMRI may be targeted with TRUS biopsy. Cognitive registration (Fig. 1) requires the TRUS operator to mentally integrate mpMRI findings with TRUS to target the lesion, which works well for large anterior tumours, but is not as accurate as an in-bore MR-guided biopsy in case of small lesions [108, 109]. Difficulties of merging mpMRI with TRUS may be overcome with the newly developed MRI-TRUS fusion software, but this also has limitations including mainly errors in fusion due to spatial deformation of the prostate at TRUS compared to MRI, and it is expensive and only available in few specialized centres [109]. Suspicious lesions detected on mpMRI may not be perfectly matched with TRUS-guided biopsy, and this must be taken into account when faced with a negative histopathological result [109]. MpMRI-guided biopsy has a significantly higher PC detection rate and positive core rate as compared to standard random TRUS-guided biopsy or extended systematic biopsies, especially in patients with previous negative biopsy [89, 106, 107, 110–114]. MpMRI is often able to detect the most aggressive PC focus, but synchronous non-index tumour detection may be poor. Therefore, it remains to be clarified whether it is safe to exclusively biopsy the target lesion while abandoning additional routine systematic biopsies or whether the target lesion should only be biopsied at a higher sampling density as compared to the rest of the tissue [97, 114].

## Defer biopsy when mpMRI is negative

**Fig. 2:**
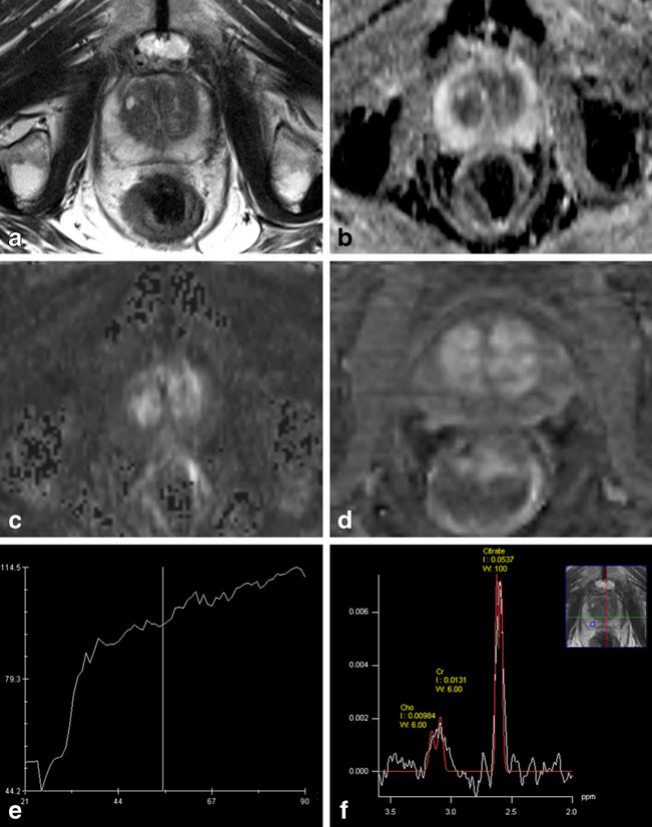
65-year-old man with PSA of 3.2 μg/l. On T2-WI (**a**) the PZ shows normal high SI. On DWI, the ADC values in the PZ are high (**b**) and the SI on the b-1000 images is low (**c**). On DCE the PZ shows no suspicious contrast enhancement (**d**) and the DCE curves show a linear pattern (**e**). On MRSI (f) the spectra show normal high citrate peaks and low choline concentrations. The overall conclusion of this mpMRI exam was PI-RADS1. On the basis of this mpMRI, a clinically significant PC could be excluded with high certainty. A biopsy may reasonably be deferred, or if a biopsy shows low grade PC in a few cores, this patient is a good candidate for active surveillance

In men with normal findings on mpMRI (PI-RADS overall assessment score 1 or 2), the risk of a clinically significant PC is very low. Negative predictive values (NPV) of 63-91 % are reported for PC of any grade, and 92-100 % for clinically significant PC (depending on the definition of clinically significant disease used) in low risk men (PSA <10, DRE normal, no family history) [13, 28, 59, 69–75, 81, 101, 103]. Since the growth and stage progression of PC tend to be slow, consideration could, therefore, reasonably be given to deferring or even omitting a biopsy in these patients, as long as there is continued monitoring with repetitive PSA sampling, DRE, and/or mpMRI [13, 22, 99, 102, 103, 115] (Fig. 2). MpMRI is negative in men with elevated PSA in 18-33 %, indicating the potential number of patients in whom a biopsy could be avoided [13, 67, 68, 99, 103, 115, 116]. Postponing a biopsy may however hold a risk of missing or delaying a diagnosis of PC, although the majority of the missed PC on mpMRI seem to be low grade and organ-confined [116]. The frequency of missed clinically significant PC on mpMRI has been reported to be 9-13 % in a low risk group (PSA < 10 and DRE normal) but 47-51 % in a high risk group (PSA > 10 and/or DRE abnormal) [103, 116], thus, in the high risk group the role of pre-biopsy mpMRI might be limited. Missing clinically insignificant PC may be regarded as an advantage rather than a drawback, given the harmful effects of overtreatment of indolent disease. If it is anyhow detected, unnecessary treatment should be avoided, which is actually the general basis concept of active surveillance [22]. A useful way to prevent identification of insignificant disease and overtreatment for early PC might be for it not to be diagnosed histopathologically, therefore surveillance without biopsy, but repetitive mpMRI instead may be an option, and it is likely that patients would prefer a mpMRI rather than a biopsy [103].

## The indeterminate mpMRI

The findings on mpMRI may be doubtful, e.g., in case of heterogeneous low SI in the PZ on T2-WI, which may represent prostatitis but may hide or mimic PC, or in case of a small lesion in the TZ, with a background of benign prostatic hyperplasia. In the PI-RADS system these findings are assigned an overall assessment score 3, and in most publications this has been considered as a positive signal to biopsy, although this decision should not depend on the images alone. The clinician should take into account a balance of factors such as PSA level, age, comorbidity, competing mortality risk assessment, and psychological factors [68]. When the other clinical parameters are suggestive of significant PC, a patient with elevated PSA and indeterminate mpMRI should have a biopsy, but if the risk of a significant PC is estimated to be rather low, the patient may benefit from follow-up PSA and/or repeat mpMRI in 6 to 12 months [71].

## Conclusion

MpMRI may be used as an additional parameter next to PSA in the early detection of clinically significant PC. The accuracy of mpMRI for detecting clinically significant PC varies with the definition of clinically significant disease and the mpMRI threshold that is used. Pre-biopsy mpMRI improves the accuracy of a prostate biopsy by targeting suspicious lesions. In low-risk patients with elevated PSA but a negative mpMRI, consideration could be given to surveillance rather than immediate biopsy.
